# Clinical Risk Constellations for the Development of Bipolar Disorders

**DOI:** 10.3390/medicina57080792

**Published:** 2021-07-31

**Authors:** Eva Burkhardt, Andrea Pfennig, Karolina Leopold

**Affiliations:** 1 Department of Psychiatry, Psychotherapy and Psychosomatic Medicine, Vivantes Klinikum Am Urban and Vivantes Klinikum Im Friedrichshain, Teaching Hospitals of Charité-Universitätsmedizin Berlin, 10967 Berlin, Germany; eva.burkhardt@gmx.net; 2 Department of Psychiatry and Psychotherapy, Universitätsklinikum Carl Gustav Carus, Technische Universität Dresden, 01307 Dresden, Germany; andrea.pfennig@uniklinikum-dresden.de

**Keywords:** bipolar disorder, risk factors, early recognition, affective disorders

## Abstract

The early recognition of psychiatric disorders has been a focus of research in the last decades and has led to improvements in clinical care, especially in the area of early psychosis. Like non-affective psychosis, bipolar disorders are often diagnosed with a delay that can lead to long periods of untreated illness and impact long-term outcomes. This article presents the rationale for early recognition in bipolar disorder and presents the current evidence for the identification of risk factors, their assessment and validity in predicting the onset of bipolar disorder.

## 1. Importance of Early Recognition of Bipolar Disorders

Bipolar disorder is often diagnosed with a long latency. Up to ten years lie between a first episode and the start of adequate treatment [1,2]. Research in the last decades has shown that bipolar disorders, like non-affective psychoses, are often preceded by a prodromal state where people experience attenuated symptoms and impaired functioning, but the criteria for bipolar disorder are not yet fulfilled [3,4,5,6]. A multitude of reasons contributes to the delayed confirmation of diagnosis and treatment. Early symptoms of bipolar disorder often emerge during adolescence and the first full-blown depressive and (hypo-)manic episodes usually occur in or before early adulthood [7]. Young people are often hesitant to seek help for mental health difficulties due to limited awareness, the stigma of mental illness and structural deficits of support services in engaging with this population [8,9]. The typical course of bipolar disorder where episodes of mood symptoms are interrupted by periods with significantly fewer or no symptoms is sometimes difficult to distinguish from age-appropriate changes during puberty, like a shifting circadian rhythm and mood swings. Especially hypomanic episodes are often experienced as somewhat pleasant and not as problematic, or a sign of mental illness by the person themselves. In about half of the cases, bipolar disorder manifests with a depressive episode first. If this is the case, or if people predominantly experience depressive episodes, they are usually diagnosed with unipolar disorder and brief periods of hypomania can easily be missed in the clinical assessment [10,11].

Long periods of untreated illness are associated with more severe symptoms, a higher number of episodes, longer periods with depressive symptoms, shorter or less euthymic phases and rapid cycling [12]. Furthermore, treatment delay can lead to poorer functional outcomes, elevated risk of suicide, longer or more frequent hospitalizations, and poor response to mood-stabilizing medication [13,14,15,16]. To improve outcomes, clinical services need to facilitate early recognition of bipolar disorder and, ideally, the identification of people at risk for the development of the disorder to offer tailored support appropriate for the stage of illness [17,18]. As bipolar disorder often manifests during adolescence and early adulthood, it can interrupt a crucial developmental period and cause long-term social and occupational difficulties, like delayed or failed completion of education and difficulty in forming lasting friendships or romantic relationships. Early intervention should aim to not only reduce symptoms, but also improve psychosocial functioning, by potentially preventing or delaying the onset of bipolar disorder and supporting young people and their families to effectively manage the risk of future episodes [19,20,21].

## 2. Exploring Risk Factors and Recognizing Prodromal States of Bipolar Disorder

Bipolar disorder is considered a highly heritable disorder, although the genetic mechanism of the inherited risk remains largely unknown [22]. Bipolar disorder in a parent, especially with manifestation at a younger age, is considered the most important risk factor for bipolar disorder [23]. Prospective studies in young people with a family history of bipolar disorder have aimed to improve the understanding of different trajectories and identify prodromal symptoms [3,24]. The risk of developing bipolar disorder for offspring of parents with bipolar disorder is increased 10- to 15-fold, and they often experience a prodromal phase with reduced psychosocial functioning, subclinical affective symptoms (labile mood, attenuated depressive and hypomanic features) and nonspecific symptoms like sleep disturbances, anxiety and substance use [4,24,25,26]. It is unclear if these results are generalizable to people without genetic risk [24]. However, family history of bipolar disorder can only explain part of the variability in risk for the disorder [27] and family history of major depression, schizophrenia or schizoaffective disorder also increases the risk of developing bipolar disorder as these conditions appear to have strong genetic correlations [22,28,29].

Similar to schizophrenia-spectrum disorders, bipolar disorders are a heterogenic diagnostic group with high variability in severity and clinical symptoms, as well as long-term trajectory and treatment response. Therefore, research from different angles is needed to complement the findings from studies in populations with increased genetic risk. One approach has been to transfer the ultra high-risk (UHR) concept for psychosis to bipolar disorder, by defining and describing attenuated symptoms which can be weighed and combined to describe at-risk states [10,30,31]. Bechdolf et al. developed the Bipolar-At-Risk (BAR) criteria based on retrospective analyses of symptom constellations of people presenting to the UHR clinic of an early psychosis service before being diagnosed with bipolar disorder later on [30]. The Semistructured Interview for Bipolar At-Risk States (SIBARS) was designed and evaluated to assess the family history and clinical symptoms as defined in the BAR criteria, including subclinical or full-blown depression in combination with attenuated or very brief (hypo)manic symptoms or cyclothymia [32]. The described symptoms resemble bipolar disorder but do not meet the diagnostic threshold in terms of intensity, duration or impact on the level of functioning. In parallel, Correll et al. developed and validated the Bipolar Prodrome Symptom Scale (BPSS) as a structured tool for the assessment of attenuated mania [31].

**Figure 1:**
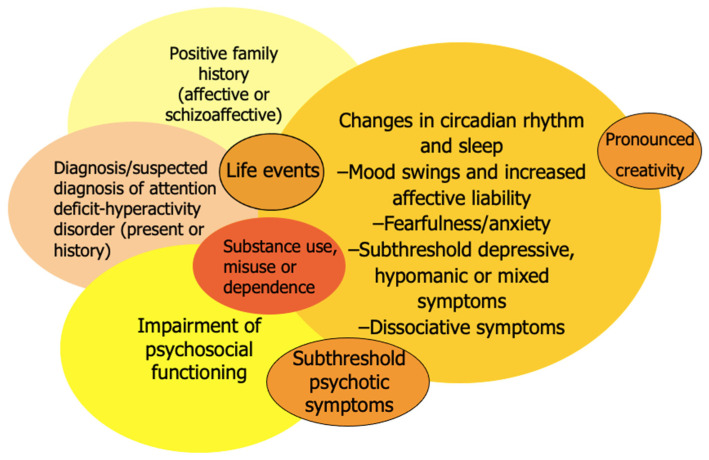
Risk constellations for bipolar disorders.

The BAR criteria and BPSS both focus on subclinical affective symptoms based on the assumption that those symptoms could increase in severity, duration or frequency and eventually meet the threshold for a diagnosis of bipolar disorder. The semistructured Early Phase Inventory for Bipolar Disorder (EPI*bipolar*) aims to improve sensitivity and specificity by combining this symptom-oriented approach with the evaluation of non-affective symptoms or risk factors that might be present in euthymic periods and even before the first manifestations of affective symptoms [10]. These risk factors include changes in sleep and circadian rhythm [33,34,35], specific patterns of substance use [36,37,38], childhood anxiety disorder [39], ADHD [40] and pronounced creativity [41,42] (see Figure 1).

## 3. Broadening the Scope for Risk Constellations in Mental Health

While many lessons have been learned from diagnosis-specific research on risk factors and prodromal symptoms, some have argued for a model of a broad high-risk syndrome, a pluripotential clinical state that may later differentiate into specific diagnostic entities [43,44]. Given the heterogeneity of presentations within diagnostic groups like schizophrenia-spectrum psychosis, affective disorders, personality disorders, anxiety, OCD and many others, as well as the overlap between these categories and frequent comorbidity, the attempt to class unspecific and attenuated symptoms as a distinct high-risk syndrome for a single diagnostic group appears arbitrary. Most risk factors are shared between different disorders—for example, traumatic life events (especially childhood trauma), substance use, anxiety symptoms and poor psychosocial functioning. 

Nevertheless, early diagnostic clarification remains important to facilitate a tailored treatment approach—for example, the initiation of a mood-stabilizing medication for people meeting criteria for bipolar disorder or prioritization of evidence-based psychotherapy (e.g., dialectic behavioral therapy) for young people with mood swings in the context of borderline personality disorder. To reliably differentiate between bipolar disorder and unipolar depression or schizophrenia-spectrum disorders, it is necessary to identify risk factors that have a higher specificity for bipolar disorder. Promising candidates might be periodic disturbances of sleep or circadian rhythm and creativity. The latter does not appear to be a symptom of bipolar disorder but a trait associated with certain symptoms (e.g., cyclothymia) or personality features that may, in turn, be linked to bipolar disorder [42,45]. Relatives of people with bipolar disorder show higher rates of creativity [46,47], and genetic links between bipolar disorder and creativity have been identified [22]. Against this background, it seems plausible that creativity as a trait could be present before the onset of bipolar disorder.

## 4. Current Evidence for At-Risk States for Bipolar Disorder

To date, three structured, standardized measures for at-risk states of bipolar disorder have been published: the Bipolar Prodrome Symptom Scale—Prospective (BPSS-P) [48], the Semistructured Interview for Bipolar At Risk States (SIBARS) [32] and the Early Phase Inventory for Bipolar Disorders (EPI*bipolar*) [10]. The BPSS showed good reliability, internal consistency and convergent validity [31] and has so far been evaluated with retrospective data [49]. In contrast, a prospective evaluation detailing specificity, sensitivity and prognostic validity is not yet available. In initial studies in Australia, without the application of the later designed structured assessment tool SIBARS, 22.8% of young people meeting BAR criteria experienced a first episode of (hypo)mania (mostly within a year after the initial assessment) in a retrospective analysis [30] and 14.3% developed (hypo)mania within twelve months in a prospective study [50]. The first study using the slightly modified Bipolar-At-Risk-States (BARS) criteria and the SIBARS measure observed transition to bipolar disorder in 23.4% of the BARS-positive individuals within two years and an adequate prognostic accuracy [32]. 

The abovementioned transition rates to bipolar disorder may appear relatively low compared to those in early studies of UHR cohorts where up to 30–40% developed first-episode psychosis within a year [51,52,53]. However, transition rates have been declining in UHR studies in recent years, likely due to less delay in people being referred to specialized early detection and early intervention centers and improved clinical care for young people in these services [54,55,56]. In addition, the course of bipolar disorder is typically episodic with long euthymic phases or periods with only mild symptoms. Up to ten years lie between the first symptoms of bipolar disorder and the manifestation of the full-blown disorder with at least one (hypo)manic and depressive episode [2]. Considering this, studies examining transition risk in people with attenuated or brief affective symptoms and/or genetic risk likely require follow-up periods of longer than 12 months. Through the combination of non-affective symptoms and risk factors and a detailed assessment of frequency and patterns of occurrence for each symptom, EPI*bipolar* aims for a higher predictive validity [10,57]. The validation of EPI*bipolar* as part of the multicenter study BipoLife, intending to follow up the initial cohort of 1419 young people for at least two years, is ongoing [58].

## 5. Ethical Considerations

Early recognition and early intervention approaches for any medical condition always require careful balancing of potential risks and benefits for the individual as well as the cost–benefit ratio from a public health perspective. The delay of ineffective treatment of people with bipolar disorder not only contributes to a diminished quality of life and poor long-term outcomes for the affected person but also cause significant direct and indirect societal costs, mainly in the healthcare system [59,60]. Preventive strategies such as early recognition and tailored interventions for bipolar disorder are needed to mitigate the potential negative consequences of the disorder for individuals, their families and society.

As pointed out before, bipolar disorder usually manifests during adolescence and early adulthood. Early recognition services must adapt to the needs of young people and support them and their families in making informed decisions about their mental health care. Clinicians should be aware of normal developmental changes in this phase of life and avoid pathologizing common features like a slight shift in circadian rhythm, mood swings, or changes in activity patterns, especially if they are not causing distress to the young person.

It is important to keep in mind that only a proportion of people with an at-risk status will eventually transition to bipolar disorder. A significant number of people will not develop a manifest disorder even without any preventive measures. The confrontation with the assessment as being at elevated risk to develop a severe psychiatric disorder can be distressing and potentially cause anxiety, adjustment difficulties, or a worsening of pre-existing symptoms. Conversations about diagnoses or risk constellations need to address these concerns, weighing the benefits of being informed about a potential risk (e.g., learning about warning signs of worsening symptoms, getting to know available supports) and the difficulties associated with an uncertain prognosis and the prevalent stigma of mental health difficulties. Assessments with the aim of identifying at-risk states should only be performed with a clear indication because of stressful symptoms and/or impairment of psychosocial functioning, and informed consent of the young person.

## 6. Conclusions and Future Directions

Several risk factors for bipolar disorders have been identified and partly validated in clinical studies. The different approaches need to be compared and their predictive validity should be confirmed in larger samples with longer follow-up periods. It is yet unclear how these separate risk factors interact. Some might be independent while others are closely linked through shared clinical or genetic features. Potentially, the increasing knowledge about polygenic risk scores [61] and digital sources of information, such as smartphone-based objective-monitoring data [62], could be combined with clinical assessments to increase the predictive validity of risk models.

Once our understanding of threshold effects, as well as the additive or multiplicative effects of individual factors on the overall risk improves, it might become possible to refine at-risk criteria and assessment tools, so they can more easily be applied by clinicians and inform clinical decision-making. Further research should also explore potential protective factors, e.g., social support networks [63], which could help to optimize treatment options for those at-risk. 

Bipolar disorder might be the common outcome of different clinical pathways starting from distinct risk constellations. We know that symptoms in the early phase of bipolar disorder, as well as other psychiatric disorders, are dynamic and constantly evolving [64]. Long-term observational studies with large cohorts will be needed to disentangle the complex trajectories from at-risk states to prodromal phases and manifest disorder. To ensure that at-risk mental states and early stages of psychiatric disorders are identified with minimal delay, such studies should be conducted in established specialized early recognition and early intervention services that are easily approachable for young people with emerging mental health problems. Such services, especially with a broad diagnostic spectrum, not limited to ultra-high risk states for psychosis, are still not widely available for a number of reasons, including funding difficulties, a limited number of clinicians qualified to perform assessments of at-risk mental states, and concerns around over-diagnosing and over-treatment of young people with nonspecific symptoms. International cooperation in the area of early recognition and early intervention and shared diagnostic standards are fundamental to achieve the goal of recognizing emerging mental health problems early and with high specificity, and over time, improve outcomes for young people at-risk.

## References

[1] Baldessarini R.J., Tondo L., Hennen J. (2003). Treatment-latency and previous episodes: Relationships to pretreatment morbidity and response to maintenance treatment in bipolar I and II disorders. Bipolar Disord..

[2] Pfennig A., Jabs B., Pfeiffer S., Weikert B., Leopold K., Bauer M. (2011). Versorgungserfahrungen Bipolarer Patienten in Deutschland. Nervenheilkunde.

[3] Duffy A., Alda M., Hajek T., Sherry S.B., Grof P. (2010). Early stages in the development of bipolar disorder. J. Affect. Disord..

[4] Duffy A., Alda M., Crawford L., Milin R., Grof P. (2007). The Early Manifestations of Bipolar Disorder: A Longitudinal Prospective Study of the Offspring of Bipolar Parents. Bipolar Disord..

[5] Hillegers M.H., Reichart C.G., Wals M., Verhulst F.C., Ormel J., Nolen W.A. (2005). Five-year Prospective Outcome of Psychopathology in the Adolescent Offspring of Bipolar Parents. Bipolar Disord..

[6] Reichart C.G., van der Ende J., Wals M., Hillegers M.H., Nolen W.A., Ormel J., Verhulst F.C. (2005). The Use of the GBI as Predictor of Bipolar Disorder in a Population of Adolescent Offspring of Parents with a Bipolar Disorder. J. Affect. Disord..

[7] Carlson G., Bromet E., Sievers S. (2000). Phenomenology and Outcome of Youth and Adult Onset Subjects with Psychotic Mania. Am. J. Psychiatry.

[8] Zachrisson H.D., Rödje K., Mykletun A. (2006). Utilization of Health Services in Relation to Mental Health Problems in Adolescents: A Population Based Survey. BMC Public Health.

[9] McGorry P.D., Goldstone S.D., Parker A., Rickwood D., Hickie I. (2014). Cultures for mental health care of young people: An Australian blueprint for reform. Lancet Psychiatry.

[10] Leopold K., Ritter P., Correll C.U., Marx C., Özgürdal S., Juckel G., Bauer M., Pfennig A. (2012). Risk constellations prior to the development of bipolar disorders: Rationale of a new risk assessment tool. J. Affect. Disord..

[11] Faedda G.L., Baldessarini R.J., Marangoni C., Bechdolf A., Berk M., Birmaher B., Conus P., DelBello M.P., Duffy A.C., Hillegers M.H. (2019). An International Society of Bipolar Disorders Task Force Report: Precursors and Prodromes of Bipolar Disorder. Bipolar Disord..

[12] Post R.M., Leverich G.S., Kupka R.W., Keck Jr P.E., McElroy S.L., Altshuler L.L., Frye M.A., Luckenbaugh D.A., Rowe M., Grunze H. (2010). Early-Onset Bipolar Disorder and Treatment Delay Are Risk Factors for Poor Outcome in Adulthood. J. Clin. Psychiatry.

[13] Goldberg J.F., Ernst C.L. (2002). Features Associated With the Delayed Initiation of Mood Stabilizers at Illness Onset in Bipolar Disorder. J. Clin. Psychiatry.

[14] Vedel K.L., Eleni V., Andersen P.K. (2014). Starting Lithium Prophylaxis Early v. Late Inbipolar Disorder. Br. J. Psychiatry.

[15] Miller S., Dell’Osso B., Ketter T.A. (2014). The Prevalence and Burden of Bipolar Depression. J. Affect. Disord..

[16] Matza L.S., Rajagopalan K.S., Thompson C.L., De Lissovoy G. (2005). Misdiagnosed Patients with Bipolar Disorder: Comorbidities, Treatment Patterns, and Direct Treatment Costs. J. Clin. Psychiatry.

[17] Forsman A.K., Wahlbeck K., Aarø L.E., Alonso J., Barry M.M., Brunn M., Cardoso G., Cattan M., de Girolamo G., Eberhard-Gran M. (2015). Research priorities for public mental health in Europe: Recommendations of the ROAMER project. Eur. J. Public Health.

[18] Berk M., Post R., Ratheesh A., Gliddon E., Singh A., Vieta E., Carvalho A.F., Ashton M., Berk L., Cotton S. (2017). Staging in bipolar disorder: From theoretical framework to clinical utility. World Psychiatry.

[19] Rios A.C., Noto M.N., Rizzo L.B., Mansur R., Martins Jr F.E., Grassi-Oliveira R., Correll C.U., Brietzke E. (2015). Early Stages of Bipolar Disorder: Characterization and Strategies for Early Intervention. Braz. J. Psychiatry.

[20] Berk M., Hallam K., Lucas N., Hasty M., McNeil C.A., Conus P., Kader L., McGorry P.D. (2007). Early Intervention in Bipolar Disorders: Opportunities and Pitfalls. Med. J. Aust..

[21] Correll C.U., Penzner J.B., Frederickson A.M., Richter J.J., Auther A.M., Smith C.W., Kane J.M., Cornblatt B.A. (2007). Differentiation in the Preonset Phases of Schizophrenia and Mood Disorders: Evidence in Support of a Bipolar Mania Prodrome. Schizophr. Bull..

[22] Gordovez F.J.A., McMahon F.J. (2020). The genetics of bipolar disorder. Mol. Psychiatry.

[23] Hafeman D.M., Merranko J., Axelson D., Goldstein B.I., Goldstein T., Monk K., Hickey M.B., Sakolsky D., Diler R.S., Iyengar S. (2016). Toward the Definition of a Bipolar Prodrome: Dimensional Predictors of Bipolar Spectrum Disorders in At-Risk Youths. Am. J. Psychiatry.

[24] Duffy A., Horrocks J., Doucette S., Keown-Stoneman C., McCloskey S., Grof P. (2014). The Developmental Trajectory of Bipolar Disorder. Br. J. Psychiatry.

[25] Hafeman D.M., Merranko J., Goldstein T.R., Axelson D., Goldstein B.I., Monk K., Hickey M.B., Sakolsky D., Diler R., Iyengar S. (2017). Assessment of a Person-Level Risk Calculator to Predict New-Onset Bipolar Spectrum Disorder in Youth at Familial Risk. JAMA Psychiatry.

[26] Axelson D., Goldstein B., Goldstein T., Monk K., Yu H., Hickey M.B., Sakolsky D., Diler R., Hafeman D., Merranko J. (2015). Diagnostic Precursors to Bipolar Disorder in Offspring of Parents with Bipolar Disorder: A Longitudinal Study. Am. J. Psychiatry.

[27] Kieseppä T., Partonen T., Haukka J., Kaprio J., Lönnqvist J. (2004). High Concordance of Bipolar I Disorder in a Nationwide Sample of Twins. Am. J. Psychiatry.

[28] Tesli M., Espeseth T., Bettella F., Mattingsdal M., Aas M., Melle I., Djurovic S., Andreassen O. (2014). Polygenic Risk Score and the Psychosis Continuum Model. Acta Psychiatr. Scand..

[29] Purcell S.M., Wray N.R., Stone J.L., Visscher P.M., O’Donovan M.C., Sullivan P.F., Sklar P., Purcell (Leader) S.M., Stone J.L., Sullivan P.F. (2009). Common Polygenic Variation Contributes to Risk of Schizophrenia and Bipolar Disorder. Nature.

[30] Bechdolf A., Nelson B., Cotton S., Chanen A., Thompson A., Kettle J., Conus P., Amminger G.P., Yung A., Berk M. (2010). A preliminary evaluation of the validity of at-risk criteria for bipolar disorders in help-seeking adolescents and young adults. J. Affect. Disord..

[31] Correll C.U., Olvet D.M., Auther A.M., Hauser M., Kishimoto T., Carrión R.E., Snyder S., Cornblatt B.A. (2014). The Bipolar Prodrome Symptom Interview and Scale–Prospective (BPSS-P): Description and Validation in a Psychiatric Sample and Healthy Controls. Bipolar Disord..

[32] Fusar-Poli P., De Micheli A., Rocchetti M., Cappucciati M., Ramella-Cravaro V., Rutigliano G., Bonoldi I., McGuire P., Falkenberg I. (2018). Semistructured Interview for Bipolar At Risk States (SIBARS). Psychiatry Res..

[33] Ritter P.S., Höfler M., Wittchen H.-U., Lieb R., Bauer M., Pfennig A., Beesdo-Baum K. (2015). Disturbed Sleep as Risk Factor for the Subsequent Onset of Bipolar Disorder–Data from a 10-Year Prospective-Longitudinal Study among Adolescents and Young Adults. J. Psychiatr. Res..

[34] Rucklidge J.J. (2008). Retrospective Parent Report of Psychiatric Histories: Do Checklists Reveal Specific Prodromal Indicators for Postpubertal-onset Pediatric Bipolar Disorder?. Bipolar Disord..

[35] Egeland J.A., Hostetter A.M., Pauls D.L., Sussex J.N. (2000). Prodromal Symptoms Before Onset of Manic-Depressive Disorder Suggested by First Hospital Admission Histories. J. Am. Acad. Child. Adolesc. Psychiatry.

[36] Angst J., Gamma A., Endrass J., Rössler W., Ajdacic-Gross V., Eich D., Herrell R., Merikangas K.R. (2006). Is the Association of Alcohol Use Disorders with Major Depressive Disorder a Consequence of Undiagnosed Bipolar-II Disorder?. Eur. Arch. Psychiatry Clin. Neurosci..

[37] Grant B.F., Stinson F.S., Dawson D.A., Chou S.P., Dufour M.C., Compton W., Pickering R.P., Kaplan K. (2004). Prevalence and Co-Occurrence of Substance Use Disorders and Independentmood and Anxiety Disorders: Results from the National Epidemiologic Survey on Alcohol and Relatedconditions. Arch. Gen. Psychiatry.

[38] Burns L., Teesson M. (2002). Alcohol Use Disorders Comorbid with Anxiety, Depression and Drug Use Disorders: Findings from the Australian National Survey of Mental Health and Well Being. Drug Alcohol Depend..

[39] Bittner A., Goodwin R.D., Wittchen H.-U., Beesdo K., Höfler M., Lieb R. (2004). What Characteristics of Primary Anxiety Disorders Predict Subsequent Major Depressive Disorder?. J. Clin. Psychiatry.

[40] Kim E.Y., Miklowitz D.J. (2002). Childhood Mania, Attention Deficit Hyperactivity Disorder and Conduct Disorder: A Critical Review of Diagnostic Dilemmas. Bipolar Disord..

[41] Thys E., Sabbe B., De Hert M. (2014). Creativity and Psychopathology: A Systematic Review. Psychopathology.

[42] Burkhardt E., Pfennig A., Breitling G., Pfeiffer S., Sauer C., Bechdolf A., Correll C.U., Bauer M., Leopold K. (2019). Creativity in Persons At-Risk for Bipolar Disorder—A Pilot Study. Early Interv. Psychiatry..

[43] McGorry P.D., Hartmann J.A., Spooner R., Nelson B. (2018). Beyond the “at Risk Mental State” Concept: Transitioning to Transdiagnostic Psychiatry. World Psychiatry.

[44] Hartmann J.A., McGorry P.D., Destree L., Amminger G.P., Chanen A.M., Davey C.G., Ghieh R., Polari A., Ratheesh A., Yuen H.P. (2021). Pluripotential Risk and Clinical Staging: Theoretical Considerations and Preliminary Data from a Transdiagnostic Risk Identification Approach. Front. Psychiatry.

[45] Akiskal K.K., Savino M., Akiskal H.S. (2005). Temperament Profiles in Physicians, Lawyers, Managers, Industrialists, Architects, Journalists, and Artists: A Study in Psychiatric Outpatients. J. Affect. Disord..

[46] Simeonova D.I., Chang K.D., Strong C., Ketter T.A. (2005). Creativity in Familial Bipolar Disorder. J. Psychiatr. Res..

[47] Kyaga S., Lichtenstein P., Boman M., Hultman C., Långström N., Landén M. (2011). Creativity and Mental Disorder: Family Study of 300,000 People with Severe Mental Disorder. Br. J. Psychiatry.

[48] Correll C.U., Auther A.M., Lencz T., Cornblatt B.A. (2005). Bipolar Prodrome Symptom Scale (BPSS).

[49] Correll C.U., Hauser M., Penzner J.B., Auther A.M., Kafantaris V., Saito E., Olvet D., Carrión R.E., Birmaher B., Chang K.D. (2014). Type and Duration of Subsyndromal Symptoms in Youth with Bipolar I Disorder Prior to Their First Manic Episode. Bipolar Disord..

[50] Bechdolf A., Ratheesh A., Cotton S.M., Nelson B., Chanen A.M., Betts J., Bingmann T., Yung A.R., Berk M., McGorry P.D. (2014). The Predictive Validity of Bipolar At-Risk (Prodromal) Criteria in Help-Seeking Adolescents and Young Adults: A Prospective Study. Bipolar Disord..

[51] Yung A.R., Yuen H.P., McGorry P.D., Phillips L.J., Kelly D., Dell’Olio M., Francey S.M., Cosgrave E.M., Killackey E., Stanford C. (2005). Mapping the Onset of Psychosis: The Comprehensive Assessment of At-risk Mental States. Aust. N. Z. J. Psychiatry.

[52] Bechdolf A., Wagner M., Ruhrmann S., Harrigan S., Putzfeld V., Pukrop R., Brockhaus-Dumke A., Berning J., Janssen B., Decker P. (2012). Preventing Progression to First-Episode Psychosis in Early Initial Prodromal States. Br. J. Psychiatry.

[53] Ruhrmann S., Schultze-Lutter F., Salokangas R.K., Heinimaa M., Linszen D., Dingemans P., Birchwood M., Patterson P., Juckel G., Heinz A. (2010). Prediction of Psychosis in Adolescents and Young Adults at High Risk: Results from the Prospective European Prediction of Psychosis Study. Arch. Gen. Psychiatry.

[54] Yung A.R., Yuen H.P., Berger G., Francey S., Hung T.-C., Nelson B., Phillips L., McGorry P. (2007). Declining Transition Rate in Ultra High Risk (Prodromal) Services: Dilution or Reduction of Risk?. Schizophr. Bull..

[55] Wiltink S., Velthorst E., Nelson B., McGorry P.M., Yung A.R. (2015). Declining Transition Rates to Psychosis: The Contribution of Potential Changes in Referral Pathways to an Ultra–High-risk Service. Early Inter. Psychiatry.

[56] Formica M., Phillips L., Hartmann J., Yung A., Wood S., Lin A., Amminger G., McGorry P., Nelson B. (2020). Has Improved Treatment Contributed to the Declining Rate of Transition to Psychosis in Ultra-High-Risk Cohorts?. Schizophr. Res..

[57] Leopold K., Ratzer S., Correll C.U., Rottmann-Wolf M., Pfeiffer S., Ritter P., Bauer M., Pfennig A. (2014). Characteristics, Symptomatology and Naturalistic Treatment in Individuals at-Risk for Bipolar Disorders: Baseline Results in the First 180 Help-Seeking Individuals Assessed at the Dresden High-Risk Project. J. Affect. Disord..

[58] Pfennig A., Leopold K., Martini J., Boehme A., Lambert M., Stamm T., Bermpohl F., Reif A., Kittel-Schneider S., Juckel G. (2020). Improving Early Recognition and Intervention in People at Increased Risk for the Development of Bipolar Disorder: Study Protocol of a Prospective-Longitudinal, Naturalistic Cohort Study (Early-BipoLife). Int. J. Bipolar Disord..

[59] Ketter T.A. (2010). Diagnostic Features, Prevalence, and Impact of Bipolar Disorder. J. Clin. Psychiatry.

[60] Kleine-Budde K., Touil E., Moock J., Bramesfeld A., Kawohl W., Rössler W. (2014). Cost of Illness for Bipolar Disorder: A Systematic Review of the Economic Burden. Bipolar Disord..

[61] Craddock N., Sklar P. (2013). Genetics of Bipolar Disorder. Lancet.

[62] Faurholt-Jepsen M., Bauer M., Kessing L.V. (2018). Smartphone-Based Objective Monitoring in Bipolar Disorder: Status and Considerations. Int. J. Bipolar Disord..

[63] Petermann U., Petermann F. (2005). Risiko-und Schutzfaktoren in der kindlichen Entwicklung. Familienpolitik und Soziale Sicherung.

[64] Nelson B., McGorry P.D., Wichers M., Wigman J.T., Hartmann J.A. (2017). Moving from Static to Dynamic Models of the Onset of Mental Disorder: A Review. JAMA Psychiatry.

